# Clinicopathological Characteristics and Prognosis of Proximal and Distal Gastric Cancer during 1997–2017 in China National Cancer Center

**DOI:** 10.1155/2019/9784039

**Published:** 2019-06-13

**Authors:** Lulu Zhao, Huang Huang, Dongbin Zhao, Chengfeng Wang, Yantao Tian, Xinghua Yuan, Fuhai Ma, Hu Ren, Yajie Zhao, Saderbiek Aimaiti, Shuisheng Zhang, Hong Zhou, Tongbo Wang, Nianchang Wang, Yuemin Sun, Xiaofeng Bai, Yingtai Chen

**Affiliations:** ^1^National Cancer Center/National Clinical Research Center for Cancer/Cancer Hospital, Chinese Academy of Medical Sciences and Peking Union Medical College, Beijing 100021, China; ^2^Department of Environmental Health Sciences, Yale University School of Public Health, New Haven, CT 06520, USA

## Abstract

**Background:**

The prognostic relevance of gastric tumor location has been reported and debated. Our study was conducted to examine the differences in clinicopathological features, prognostic factors, and overall survival (OS) between patients with proximal gastric cancer (PGC) and distal gastric cancer (DGC).

**Patients and Methods:**

Patients with PGC or DGC were identified from the China National Cancer Center Gastric Cancer Database (NCCGCDB) during 1997–2017. Survival analysis was performed via Kaplan-Meier estimates and Cox proportional hazards models.

**Results:**

We reviewed 16,119 cases of gastric cancer patients, including 6,479 of PGC and 9,640 of DGC. PGC patients presented as older patients (61.5 versus 56.4 years,* P<*0.001) and more males (82.9% versus 68.2%,* P*<0.001). Compared with DGC, PGC was more likely to be in later pT stage (pT3 and pT4, 65.0% versus 52.8%,* P*<0.001) and lymph node metastasis (54.8% versus 50.9%,* P*<0.001). In univariate analysis, PGC patients had a worse survival outcome in stage I (Hazard ratio [HR] = 2.04, 95% CI: 1.42-2.94) but a better prognosis in stage IV (HR = 0.85, 95% CI: 0.73-0.98) when compared to DGC patients. However, multivariate analysis demonstrated that PGC was not an independent predictor for poor survival (HR = 1.07, 95% CI: 1.00-1.14). Results from multivariate analysis also revealed that pT4, lymph node metastasis, distant metastasis, no gastrectomy, and Borrmann IV were independent predictors associated with poor survival for both PGC and DGC patients. Additional prognostic factors for PGC patients included underweight (BMI < 18.5) (HR = 1.29, 95% CI: 1.06-1.58), linitis plastica (HR = 2.13, 95% CI: 1.25-3.65), and overweight (23 ≤ BMI <27.5) (HR = 0.80, 95% CI: 0.71-0.90). During the 20-year study period, the 5-year OS increased significantly for both PGC and DGC, with the increase rate of 91.7% and 67.7%, respectively.

**Conclusion:**

In China, PGC significantly differed from DGC in clinicopathological characteristics and prognostic factors. However, there was no significant relationship between survival outcome and gastric tumor location.

## 1. Introduction

Gastric cancer (GC) is the third leading cause of cancer-related mortality and the fifth most common cancer globally [1]. Many population-based studies have reported that the incidence of distal gastric cancer (DGC) has gradually declined, while proximal gastric cancer (PGC) has increased obviously during the last decades [2–9].

Researches have indicated that PGC differed from DGC in clinicopathological characteristics [10–13]. For example, one previous study [11] found that PGC patients were more likely to be in an advanced tumor stage and have larger tumor size as compared to DGC. Yu et al. [13] showed that PGC was more common than DGC in males. Moreover, there was no clear agreement on the link between tumor location and overall survival (OS) of GC. Some studies [11, 13–17] reported a worse prognosis in patients with PGC compared to DGC, while others [10, 12, 18] have shown no relationship between prognosis and gastric tumor location. Katsuhiko et al. [19] even demonstrated that PGC patients had a longer survival time than DGC after chemotherapy. The inconsistent findings from these previous studies could be partially due to the small sample size, with the population records ranging from 270 to 3,193.

Given the suggested but undecided differences in clinicopathological characteristics and prognosis between PGC and DGC, the aim of our study was to compare the clinicopathological features, prognostic factors, and survival outcomes between PGC and DGC based on the China National Cancer Center Gastric Cancer Database (NCCGCDB) in order to determine whether PGC conveys worse prognosis and provides evidence for the development of guiding strategies for GC patients with different tumor locations.

## 2. Materials and Methods

### 2.1. Patient Population

**Figure 1:**
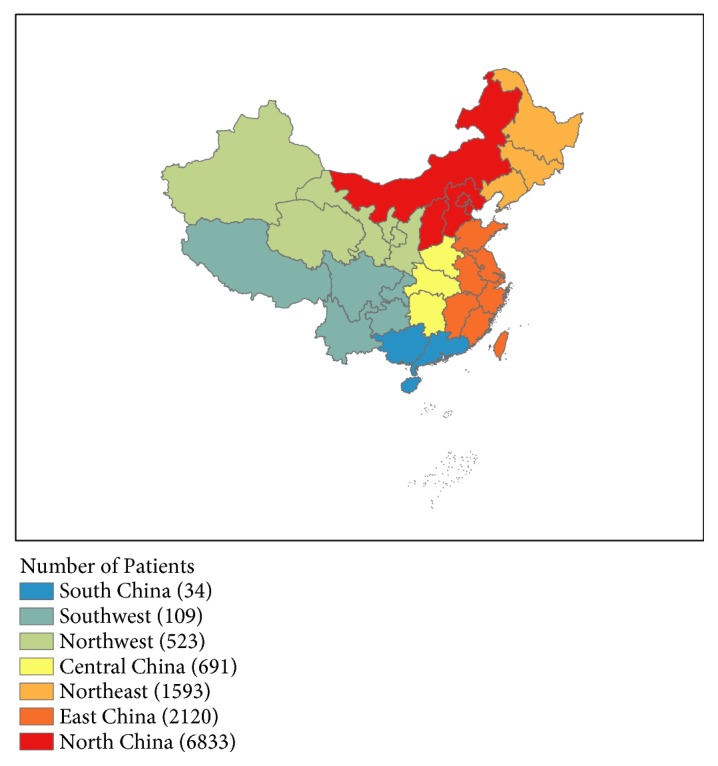
The geographical locations of PGC and DGC patients of NCCGCDB, 1997–2017.

All the study data were abstracted from the NCCGCDB. The NCCGCDB was a clinical gastric cancer database based on a huge retrospective cohort, which was sourced from China National Cancer Center, a single but large-volume institution, and included more than 19,000 patients from all around China from 1997 to 2018. PGC was defined as tumors with the epicenter located in cardia (C16.0) or fundus (C16.1), whereas DGC was defined as lesions of the body (C16.2), antrum (C16.3), or pylorus (C16.4). Changing trends in clinicopathological characteristics and OS of total GC, PGC, and DGC were analyzed in four consecutive time periods: from 1997 to 2002 (period 1), from 2002 to 2007 (period 2), from 2007 to 2012 (period 3), and from 2012 to 2017 (period 4). The geographical locations of these gastric cancer patients can be found in Figure 1.

### 2.2. Statistical Analyses

Categorical variables were compared using the Chi-squared test and continuous variables were analyzed by Student's* t*-test. OS and progression-free survival (PFS) curves were plotted for PGC and DGC groups, respectively, using the Kaplan-Meier method and compared statistically using the log-rank test. Hazard ratios (HRs) and 95% confidence intervals (CIs) were used to estimate the risk of death by employing the multivariate Cox proportional hazards models with adjustment for alcohol consumption, BMI,* H. pylori* infection, pT stage, pN stage, pM stage, Lauren classification, gastrectomy, surgical margin, HER2 score, linitis plastica, Borrmann classification, and gross classification. The covariates included in the final models were selected by the stepwise selection method, with a significant level for adding variables of 0.05 and a significant level for removing variables of 0.10. A two-sided* P* value less than 0.05 was considered as statistically significant. All the statistical analyses were performed using SAS software v9.4 (SAS Institute, Inc., Cary, NC).

## 3. Results

### 3.1. Clinicopathological Characteristics

In this study, 16,119 patients were included. The clinicopathological features of 9,640 patients (59.8%) with DGC and 6,479 patients (41.2%) with PGC were compared (Table 1), with an incidence of DGC:PGC = 1.49:1. Among our study population, a higher tumor incidence was found in DGC. There were significant differences in the distribution of age, gender, smoking, alcohol consumption, BMI,* H. pylori* infection, pTNM stage, Lauren classification, surgical margin, HER2 score, linitis plastica, and Borrmann classification between DGC and PGC patients. PGC was more likely to occur in older patients (61.5 versus 56.4 years,* P<*0.001). Both groups were predominantly males and PGC has a greater proportion of males than DGC (82.9% versus 68.2%,* P*<0.001). Relatively higher percentages of smokers (51.7% versus 33.9%,* P*<0.001), alcohol drinkers (41.7% versus 29.0%,* P*<0.001), and overweight/obesity (BMI≥23) (56.6% versus 51.2%,* P*<0.001) were shown in PGC patients as compared to DGC patients.

As for tumors, PGC patients were more likely to be in later pT stage (pT3 and pT4, 65.0% versus 52.8%,* P*<0.001), lymph node metastasis (54.8% versus 50.9%,* P*<0.001), intestinal type (18.8% versus 12.2%,* P*<0.001), local advanced GC (76.2% versus 65.9%,* P*<0.001), and Borrmann I (11.0% versus 4.6%,* P*<0.001). The percentages of ever received surgical treatment (81.2% versus 82.3%,* P*=0.068) were similar between the two groups. DGC patients were more common in diffuse type (17.1% versus 8.6%,* P*<0.001), early stage GC (21.5% versus 11.3%,* P*<0.001), and distant metastasis (12.8% versus 10.1%,* P*<0.001).

Changing trends of clinicopathological features in GC patients were analyzed. The proportion of pT1 tumors increased gradually with time, from 9.5% in period 1 to 22.0% in period 4, whereas the proportion of pT4 had declined from 66.0% in period 1 to 28.1% in period 4. The proportion of patients with pN0 increased from 24.5% in period 1 to 33.5% in period 4, whereas patients with pN3 were gradually decreased from 26.3% in period 1 to 21.5% in period 4. The proportion of pM1 remained relatively stable (from 11.2% to 10.7%) during the past 20 years. In pTNM stage, a significant increase was observed in stages I and II (from 12.0% and 3.8% in period 1 to 24.9% and 17.6% in period 4, resp.), while the proportion of stage III had declined from 63.0% in period 1 to 37.2% in period 4.

### 3.2. Prognostic Factors of Survival in Univariate and Multivariate Analyses

As shown in Table 2, univariate analyses of survival revealed significantly different survival based on the following parameters: overweight/obesity (BMI ≥ 23),* H. pylori* infection, advanced pT, pN, pM, and pTNM stage, Lauren classification, no gastrectomy, surgical margin, linitis plastic, and Borrmann IV for both PGC and DGC groups. For patients with PGC, additional parameters including middle and older age (HR = 0.47, 95% CI: 0.33-0.65; HR = 0.51, 95% CI: 0.37-0.72, resp.) and HER2 score of 1(+) and 2(++) (HR = 0.70, 95% CI: 0.57-0.86; HR = 0.69, 95% CI: 0.53-0.89, resp.), while smoking (HR = 0.85, 95% CI: 0.78-0.93) and alcohol drinking (HR = 0.85, 95% CI: 0.77-0.94) were additional prognostic factors for DGC patients.

The univariate analysis found a survival benefit in patients with DGC (HR = 0.87, 95% CI: 0.82-0.93). After stratification by pTNM stage, further comparison between the two groups showed that, compared to patients with DGC, PGC patients had a worse survival outcome in stage I (HR = 2.04, 95% CI: 1.42-2.94) but a better prognosis in stage IV (HR = 0.85, 95% CI: 0.73-0.98). There was no significant survival difference in stages II and III (*P=*0.84 and 0.58, resp.). However, the multivariate analysis demonstrated that PGC was not an independent predictor for poor survival (HR = 1.07, 95% CI: 1.00-1.14).

When appropriate significant factors were taken into consideration, multivariate analysis (Table 3) revealed that pT4, lymph node metastasis, distant metastasis, no gastrectomy, and Borrmann IV were independent predictors for poor prognosis in both PGC and DGC patients. Additional factors associated with increased mortality in PGC patients included underweight (BMI < 18.5) (HR = 1.29, 95% CI: 1.06-1.58) and linitis plastica (HR = 2.13, 95% CI: 1.25-3.65). Overweight (23 ≤BMI < 27.5) was a prognostic factor associated with favorable survival outcomes only for PGC (HR = 0.80, 95% CI: 0.71-0.90). In DGC group, additional factors for poor prognosis were* H. pylori* infection (HR = 1.52, 95% CI: 1.11-2.07), diffuse subtype (HR = 1.32, 95% CI: 1.04-1.67), and positive on proximal or distal margin (HR = 1.67, 95% CI: 1.16-2.41; HR = 1.57, 95% CI: 1.13-2.17, resp.). Alcohol drinkers, including current drinkers and ex-drinkers, showed better survival for DGC (HR = 0.90, 95% CI: 0.81-0.99, HR = 0.72, 95% CI: 0.56-0.93, resp.).

### 3.3. Changing Trends of OS and PFS for Patients with PGC and DGC

**Figure 2:**
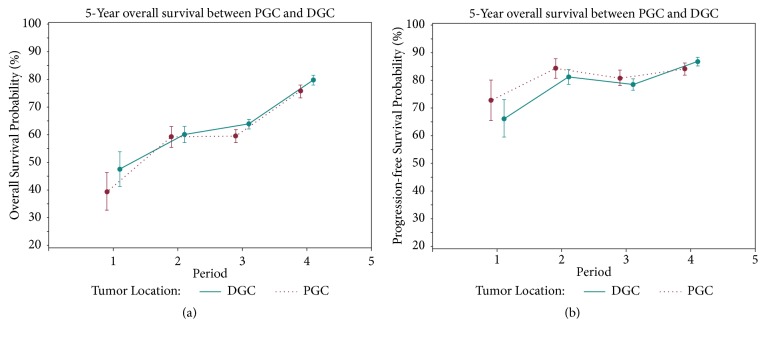
(a) The changing trends of 5-year OS of PGC and DGC from period 1 to period 4. (b) The changing trends of PFS of PGC and DGC from period 1 to period 4.

The changing trends of 5-year OS and PFS for PGC and DGC patients were shown in Figure 2(a). The total 5-year OS for GC, PGC, and DGC was 66.5% (95% CI: 65.5%-67.4%), 63.9% (95% CI: 62.4%-65.5%), and 68.1% (95% CI: 66.9%-69.3%), respectively. For total GC, 5-year OS increased from 44.1% (95% CI: 39.4%-48.7%) in period 1 to 78.4% (95% CI: 77.0%-79.7%) in period 4. The 5-year OS of PGC and DGC rose from 39.6% (95% CI: 32.8%-46.4%) to 75.9% (95% CI: 73.6%-78.1%) and from 47.7% (95% CI: 41.4%-54.0%) to 80.0% (95% CI: 78.3%-81.7%) during the 20-year study period, respectively.

There was also an increase in PFS of PGC and DGC groups during the 20 years (Figure 2(b)). The total PFS for GC, PGC, and DGC was 82.0% (95% CI: 81.1%-82.9%), 82.3% (95% CI: 80.9%-83.8%), and 81.9% (95% CI: 80.7%-83.0%), respectively. The PFS of PGC and DGC in period 1 was 72.9% (95% CI: 65.5%-80.3%) and 66.2% (95% CI: 59.4%-73.0%), respectively, while the PFS of PGC and DGC in period 4 was 84.2% (95% CI: 82.1%-86.3%) and 86.9% (95% CI: 85.3%-88.4%), respectively.

## 4. Discussion

In this study, the clinicopathological characteristics of PGC patients presented differently with DGC patients. Although two groups were predominantly males, PGC had a greater proportion of males than DGC. This was similar to some previous reports [13, 20, 21]. Yu et al. [13] reported that the gender ratio (M:F) in PGC was up to 5:1. This may be due to poor diet and unhealthy habits in men, such as smoking or alcohol consumption [22].

In addition, our study demonstrated that PGC presented to be more frequent in older patients as compared to DGC, which was similar to two published Chinese reports [13, 21]. In contrast, Park et al. from Korea [12] had shown that PGC patients were more likely to be younger. Two European studies, however, had reported no association between age and tumor location [10, 17]. These differences may be partly attributed to the genetic distinction from populations of different countries.

A primary finding of our study was that PGC was not independently associated with overall mortality, although it has long been thought to confer worse prognosis [11, 13–17]. In the univariate analysis stratified by stage, PGC patients with stage I had worse survival when compared with DGC patients, while there was no statistical survival difference between the two groups with stages II-III. However, PGC patients with stage IV had better survival than DGC. Therefore, the variations of prognosis between PGC and DGC may be related to various stage distributions existing in different studies. The reason for survival differences between PGC and DGC by stage has stayed unclear to date, and we speculate that those in tumor biology between PGC and DGC play a role.

Interestingly, the multivariate analyses reported that BMI was an independent prognostic factor for PGC patients but not for DGC patients. Moreover, a higher BMI was associated with survival benefits, while a lower BMI was associated with higher mortality, which has not been described previously. Our study also identified that no gastrectomy was an adverse independent predictor for both PGC and DGC patients, suggesting that surgery was necessary to improve survival outcomes for resected GC. Today, systematic D2 lymphadenectomy with the goal of complete (R0) resection is a generally recognized as standard surgical procedure for gastric cancer.

Our study found that 5-year survival increased significantly during the 20 years for total GC, PGC, and DGC, with an increase of 34.3%, 36.3%, and 32.3%, respectively. This was in a concord with the changing trends of increased stages I and II, as well as the decreased lymph node. Relative survival improved steadily over time for gastric cancer, suggesting an improvement in the quality of clinical services for gastric cancer patients, such as improved access to primary healthcare, greater availability of diagnostic facilities, and improved effectiveness of the multimodal treatment [23]. In China, cancer screening and early detection programs (including cancers of the esophagus, stomach, etc.) have expanded to 31 provinces until 2015 [24]. The emerging surgical procedures like endoscopic resection and laparoscopic surgery, as well as standardized procedures, also had played an important role in the prognosis of GC [25–27]. In addition, recent studies showed that the use of individually multimodal therapies had led to an improvement in the 5-year survival rate [27–29].

One limitation of this study was that it was just conducted in a single institution, so the results might not represent the whole Chinese population. However, the volume of PGC and DGC patients was large and the source of patients usually came from the area of Northern and Eastern China, which might serve as a reference for a large population-based study.

In conclusion, PGC significantly differed from DGC in clinicopathological characteristics and prognosis factors. However, there was no significant relationship between survival outcome and gastric tumor location.

## Figures and Tables

**Table 1:** Clinicopathological characteristics by tumor location.

	Total GC	PGC	DGC	*P *Value
	*N* (%)	*N* (%)	*N* (%)	
*Age at diagnosis (years)*				
Mean (SD)	58.5 (11.4)	61.5 (10.0)	56.4 (11.9)	<0.001
Younger (≤35)	590 (3.7)	86 (1.3)	504 (5.2)	
Middle-aged (36-65)	10,842 (67.3)	4,026 (62.1)	6,816 (70.7)	
Older (≥66)	4,685 (29.1)	2,366 (36.5)	2,319 (24.1)	<0.001
*Gender*				
Male	4,171 (25.9)	5,374 (82.9)	6,574 (68.2)	
Female	11,948 (74.1)	1,105 (17.1)	3,066 (31.8)	<0.001
*Smoking status*				
Never smokers	9,289 (57.6)	3,065 (47.3)	6,224 (64.6)	
Smokers	6,621 (41.1)	3,352 (51.7)	3,269 (33.9)	<0.001
Current smokers	4,634 (28.8)	2,210 (34.1)	2,424 (25.2)	
Ex-smokers	1,987 (12.3)	1,142 (17.6)	845 (8.8)	<0.001
*Alcohol consumption*				
Never drinkers	10,398 (64.5)	3,716 (57.4)	6,682 (69.3)	
Drinkers	5,496 (34.1)	2,699 (41.7)	2,797 (29.0)	<0.001
Current drinkers	4,752 (29.5)	2,416 (37.3)	2,336 (24.2)	
Ex-drinkers	744 (4.6)	283 (4.4)	461 (4.8)	<0.001
*BMI (kg/m2)*				
<18.5	1,066 (6.6)	380 (5.9)	686 (7.1)	
18.5-22.9	6,097 (37.8)	2,301 (35.5)	3,796 (39.4)	
23-27.4	6,576 (40.8)	2,760 (42.6)	3,816 (39.6)	
≥27.5	2,028 (12.6)	909 (14.0)	1,119 (11.6)	<0.001
*H. pylori*				
No	1,247 (7.7)	437 (6.7)	625 (6.5)	
Yes	956 (5.9)	331 (5.1)	810 (8.4)	
Unknown	13,916 (86.3)	5,711 (88.2)	8,205 (85.1)	<0.001
*Pathologic T stage*				
T0+Tis	58 (0.4)	14 (0.2)	44 (0.5)	
T1	2,491 (15.5)	596 (9.2)	1,895 (19.7)	
T2	1,376 (8.5)	441 (6.8)	935 (9.7)	
T3	3,019 (18.7)	1,640 (25.3)	1,379 (14.3)	
T4	6,288 (39.0)	2,573 (39.7)	3,715 (38.5)	
TX	2,887 (17.9)	1,215 (18.8)	1,672 (17.3)	<0.001
*Pathologic N stage*				
N0	4,538 (28.2)	1,623 (25.1)	2,915 (30.2)	
N1	2,281 (14.1)	983 (15.2)	1,298 (13.5)	
N2	2,417 (15.0)	1,081 (16.7)	1,336 (13.9)	
N3	3,759 (23.3)	1,489 (23.0)	2,270 (23.6)	
NX	3,124 (19.4)	1,303 (20.1)	1,821 (18.9)	<0.001
*Pathologic M stage*				
M0	13,629 (84.6)	5,555 (85.7)	8,074 (83.8)	
M1	1,883 (11.7)	651 (10.1)	1,232 (12.8)	<0.001
*pTNM*				
0	52 (0.3)	13 (0.2)	39 (0.4)	
I	2,989 (18.5)	825 (12.7)	2,164 (22.5)	
II	2,112 (13.1)	929 (14.3)	1,183 (12.3)	
III	7,354 (45.6)	3,272 (50.5)	4,082 (42.3)	
IV	1,883 (11.7)	651 (10.1)	1,232 (12.8)	<0.001
*Lauren classification*				
Intestinal	2,390 (14.8)	1,215 (18.8)	1,175 (12.2)	
Diffuse	2,202 (13.7)	555 (8.6)	1,647 (17.1)	
Mixed	1,486 (9.2)	592 (9.1)	894 (9.3)	
Unknown	10,041 (62.3)	4,117 (63.5)	5,924 (61.5)	<0.001
*Type of gastrectomy*				
Gastrectomy	13,190 (81.8)	5,260 (81.2)	7,930 (82.3)	
No surgery	2,929 (18.2)	1,219 (18.8)	1,710 (17.7)	0.068
*Surgical Margin*				
Negative	12,457 (77.3)	4,950 (76.4)	7,507 (77.9)	
Positive on the proximal margin	183 (1.1)	91 (1.4)	92 (1.0)	
Positive on the distal margin	197 (1.2)	74 (1.1)	123 (1.3)	
Positive on the proximal and distal margin	46 (0.3)	12 (0.2)	34 (0.4)	0.002
*HER2 score*				
0 (-)	2,850 (17.7)	1,037 (16.0)	1,813 (18.8)	
1 (+)	2,620 (16.3)	971 (15.0)	1,649 (17.1)	
2 (++)	1,082 (6.7)	466 (7.2)	616 (6.4)	
3 (+++)	522 (3.2)	275 (4.2)	247 (2.6)	
Unknown	9,045 (56.1)	3,730 (57.6)	5,315 (55.1)	<0.001
*Linitis plastica*				
No	15,670 (97.2)	6,286 (97.0)	9,384 (97.3)	
Yes	110 (0.7)	31 (0.5)	79 (0.8)	0.024
*Borrmann classification*				
Borrmann I	1,160 (7.2)	714 (11.0)	446 (4.6)	
Borrmann II	4,605 (28.6)	1,926 (29.7)	2,679 (27.8)	
Borrmann III	3,843 (23.8)	1,574 (24.3)	2,269 (23.5)	
Borrmann IV	981 (6.1)	347 (5.4)	634 (6.6)	
Unknown	1,807 (11.2)	696 (10.7)	1,111 (11.5)	<0.001

GC, gastric cancer; PGC, proximal gastric cancer; DGC, distal gastric cancer; SD, standard deviation.

**Table 2:** Univariate survival analysis by tumor location.

Prognostic Factors	PGC group (N=4,716)				DGC group (N=7,228)				PGC versus DGC			
	HR	95% CI		*P* Value	HR	95% CI		*P* Value	HR	95% CI		*P* Value
		Lower	Upper			Lower	Upper			Lower	Upper	
*Age (years)*												
Younger (≤35)	1.00				1.00				2.20	1.50	3.22	<0.001
Middle-aged (36-65)	0.47	0.33	0.65	<0.001	0.94	0.77	1.14	0.52	1.12	1.03	1.21	0.008
Older (≥66)	0.51	0.37	0.72	<0.001	1.01	0.82	1.24	0.92	1.14	1.01	1.28	0.028
*Gender*												
Male	1.00				1.00				1.16	1.08	1.25	<0.001
Female	1.04	0.91	1.19	0.55	1.07	0.98	1.17	0.12	1.13	0.98	1.30	0.097
*Smoking status*												
Never smoker	1.00				1.00				1.08	0.99	1.18	0.074
Smokers	1.00	0.91	1.10	0.98	0.85	0.78	0.93	<0.001	1.27	1.15	1.41	<0.001
Current smokers	1.03	0.92	1.14	0.63	0.89	0.81	0.99	0.023	1.25	1.11	1.40	<0.001
Ex-smokers	0.94	0.82	1.09	0.41	0.72	0.60	0.86	<0.001	1.39	1.13	1.71	0.002
*Alcohol consumption*												
Never drinkers	1.00				1.00				1.11	1.03	1.21	0.009
Drinkers	0.95	0.86	1.05	0.35	0.85	0.77	0.94	0.001	1.24	1.11	1.39	<0.001
Current drinkers	0.97	0.87	1.07	0.55	0.90	0.82	1.00	0.048	1.19	1.06	1.34	0.004
Ex-drinkers	0.82	0.62	1.07	0.14	0.58	0.45	0.74	<0.001	1.55	1.08	2.23	0.017
*BMI (kg/m2)*												
<18.5	1.24	1.02	1.51	0.033	1.12	0.96	1.32	0.16	1.33	1.06	1.68	0.016
18.5-22.9	1.00				1.00				1.21	1.09	1.34	<0.001
23-27.4	0.73	0.65	0.82	<0.001	0.82	0.75	0.91	<0.001	1.07	0.96	1.19	0.22
≥27.5	0.79	0.67	0.92	0.0024	0.73	0.63	0.85	<0.001	1.30	1.08	1.57	0.006
*H. pylori*												
No	1.00				1.00				1.55	1.09	2.21	0.016
Yes	1.45	1.01	2.08	0.044	1.71	1.25	2.33	0.001	1.31	0.95	1.81	0.095
Unknown	2.36	1.80	3.09	<0.001	3.31	2.60	4.21	<0.001	1.10	1.03	1.18	0.005
*Pathologic T stage*												
T0+Tis	3.28	0.79	13.58	0.10	2.58	0.94	7.08	0.066	2.46	0.45	13.53	0.30
T1	1.00				1.00				2.07	1.39	3.10	<0.001
T2	1.58	1.02	2.45	0.041	3.26	2.38	4.46	<0.001	0.96	0.67	1.38	0.84
T3	3.86	2.77	5.38	<0.001	8.32	6.39	10.83	<0.001	0.92	0.80	1.06	0.26
T4	6.69	4.83	9.28	<0.001	12.61	9.81	16.21	<0.001	1.05	0.95	1.15	0.37
TX	11.12	7.99	15.48	<0.001	27.37	21.21	35.31	<0.001	0.81	0.72	0.92	<0.001
*Pathologic N stage*												
N0	1.00				1.00				1.65	1.35	2.02	<0.001
N1	1.91	1.56	2.36	<0.001	2.39	1.96	2.90	<0.001	1.31	1.07	1.60	<0.001
N2	2.84	2.34	3.44	<0.001	3.89	3.26	4.65	<0.001	1.20	1.01	1.41	0.034
N3	5.00	4.20	5.95	<0.001	8.42	7.21	9.83	<0.001	0.97	0.86	1.08	0.56
NX	6.56	5.51	7.81	<0.001	12.60	10.79	14.73	<0.001	0.85	0.76	0.96	0.007
*Pathologic M stage*												
M0	1.00				1.00				1.28	1.18	1.38	<0.001
M1	4.96	4.33	5.68	<0.001	7.45	6.73	8.25	<0.001	0.85	0.73	0.98	0.027
*pTNM*												
0	4.06	0.99	16.71	0.052	2.25	0.71	7.14	0.17	3.16	0.53	19.05	0.21
I	1.00				1.00				2.04	1.42	2.94	<0.001
II	2.26	1.63	3.15	<0.001	4.43	3.35	5.86	<0.001	1.03	0.81	1.29	0.84
III	6.39	4.79	8.52	<0.001	12.28	9.64	15.64	<0.001	1.03	0.94	1.12	0.58
IV	23.33	17.19	31.68	<0.001	54.77	42.63	70.38	<0.001	0.85	0.73	0.98	0.027
*Lauren classification*												
Intestinal	1.00				1.00				1.68	1.31	2.15	<0.001
Diffuse	1.93	1.54	2.42	<0.001	2.09	1.66	2.62	<0.001	1.54	1.25	1.90	<0.001
Mixed	1.54	1.22	1.94	0.0003	1.37	1.04	1.81	0.024	1.88	1.45	2.46	<0.001
Unknown	2.76	2.35	3.24	<0.001	4.45	3.64	5.44	<0.001	1.05	0.97	1.13	0.22
*Stage*												
Early stage	1.00				1.00				2.09	1.31	3.32	0.002
LAGC	5.29	3.65	7.65	<0.001	9.18	7.24	13.04	<0.001	1.09	1.01	1.19	0.032
Distant	25.33	17.25	37.20	<0.001	60.25	44.52	81.52	<0.001	0.85	0.73	0.98	0.027
*Type of gastrectomy*												
Gastrectomy	1.00				1.00				1.24	1.15	1.34	<0.001
No surgery	3.01	2.71	3.35	<0.001	4.47	4.08	4.88	<0.001	0.84	0.74	0.94	0.002
*Surgical Margin*												
Negative	1.00				1.00				1.27	1.17	1.38	<0.001
Positive on the proximal margin	1.96	1.34	2.87	0.0006	2.98	2.07	4.28	<0.001	0.81	0.48	1.37	0.43
Positive on the distal margin	2.49	1.63	3.79	<0.001	2.56	1.85	3.53	<0.001	1.25	0.74	2.12	0.40
Positive on the proximal and distal margin	1.08	0.27	4.34	0.91	2.57	1.33	4.95	0.005	0.60	0.13	2.80	0.51
*HER2 score*												
0 (-)	1.00				1.00				1.45	1.22	1.73	<0.001
1 (+)	0.70	0.57	0.86	0.001	0.85	0.72	1.02	0.073	1.19	0.98	1.46	0.083
2 (++)	0.69	0.53	0.89	0.005	0.66	0.51	0.85	0.002	1.54	1.11	2.12	0.01
3 (+++)	0.94	0.71	1.26	0.68	0.99	0.72	1.38	0.96	1.38	0.93	2.07	0.11
Unknown	1.72	1.49	1.98	<0.001	2.44	2.15	2.77	<0.001	1.03	0.95	1.11	0.49
*Linitis plastica*												
No	1.00				1.00				1.15	1.08	1.23	<0.001
Yes	2.44	1.44	4.12	0.001	2.37	1.63	3.44	<0.001	1.22	0.64	2.33	0.54
*Borrmann classification*												
Borrmann I	1.00				1.00				1.03	0.78	1.35	0.85
Borrmann II	1.03	0.85	1.24	0.79	0.93	0.73	1.17	0.52	1.14	1.00	1.30	0.045
Borrmann III	1.14	0.94	1.38	0.20	1.13	0.90	1.43	0.30	1.03	0.90	1.17	0.71
Borrmann IV	2.16	1.70	2.75	<0.001	2.11	1.63	2.73	<0.001	1.05	0.84	1.31	0.67
Unknown	2.44	1.99	3.01	<0.001	3.09	2.44	3.93	<0.001	0.81	0.69	0.94	0.007
*Location*												
PGC	1.00											
DGC	0.87	0.82	0.93	<0.001								

**Table 3:** Multivariate survival analysis by tumor location.

Prognostic Factors	Total (n=11,944)				PGC group (n=4,716)				DGC group (n=7,228)			
	HR	95% CI		P Value	HR	95% CI		P Value	HR	95% CI		P Value
		Lower	Upper			Lower	Upper			Lower	Upper	
*Alcohol consumption*												
Never drinkers	1.00				1.00				1.00			
Current drinkers	0.94	0.87	1.01	0.08	0.98	0.88	1.09	0.69	0.90	0.81	0.99	0.034
Ex-drinkers	0.74	0.61	0.89	0.002	0.81	0.62	1.07	0.14	0.72	0.56	0.93	0.012
*BMI (kg/m2)*												
<18.5	1.07	0.95	1.22	0.26	1.29	1.06	1.58	0.011	0.96	0.82	1.13	0.61
18.5-22.9	1.00				1.00				1.00			
23-27.4	0.89	0.83	0.96	0.002	0.80	0.71	0.90	<0.001	0.96	0.88	1.06	0.44
≥27.5	0.91	0.82	1.01	0.09	0.94	0.80	1.09	0.41	0.87	0.75	1.01	0.07
*H. pylori*												
No	1.00				1.00				1.00			
Yes	1.43	1.13	1.81	0.003	1.35	0.94	1.94	0.11	1.52	1.11	2.07	0.009
Unknown	1.73	1.44	2.08	<0.001	1.62	1.23	2.13	<0.001	1.82	1.43	2.33	<0.001
*Pathologic T stage*												
T0+Tis	1.66	0.72	3.85	0.23	2.36	0.56	9.94	0.24	1.47	0.52	4.17	0.46
T1	1.00				1.00				1.00			
T2	1.53	1.05	2.24	0.028	0.82	0.41	1.62	0.57	1.89	1.19	3.00	0.007
T3	2.65	1.86	3.76	<0.001	1.47	0.79	2.73	0.22	3.20	2.08	4.91	<0.001
T4	3.28	2.32	4.64	<0.001	1.99	1.08	3.69	0.028	3.77	2.47	5.75	<0.001
TX	2.97	2.02	4.36	<0.001	1.46	0.76	2.81	0.26	3.88	2.42	6.23	<0.001
*Pathologic N stage*												
N0	1.00				1.00				1.00			
N1	1.36	1.17	1.59	<0.001	1.36	1.09	1.69	0.007	1.37	1.10	1.69	0.0041
N2	1.99	1.73	2.30	<0.001	1,98	1.61	2.44	<0.001	2.00	1.65	2.44	<0.001
N3	3.44	3.01	3.92	<0.001	3.29	2.70	4.01	<0.001	3.58	2.99	4.28	<0.001
NX	1.83	1.41	2.38	<0.001	1.60	1.04	2.48	0.034	1.91	1.36	2.67	<0.001
*Pathologic M stage*												
M0	1.00				1.00				1.00			
M1	3.05	2.59	3.59	<0.001	3.65	2.75	4.86	<0.001	2.84	2.32	3.48	<0.001
*Lauren classification*												
Intestinal	1.00				1.00				1.00			
Diffuse	1.22	1.03	1.43	0.018	1.21	0.95	1.55	0.12	1.32	1.04	1.67	0.024
Mixed	0.99	0.83	1.19	0.94	1.06	0.83	1.35	0.65	0.96	0.73	1.27	0.78
Unknown	1.30	1.11	1.52	<0.001	1.20	0.97	1.49	0.095	1.44	1.14	1.82	0.003
*Type of gastrectomy*												
Gastrectomy	1.00				1.00				1.00			
No surgery	1.43	1.22	1.67	<0.001	1.48	1.15	1.90	0.003	1.44	1.17	1.76	<0.001
*Surgical Margin*												
Negative	1.00				1.00				1.00			
Positive on the proximal margin	1.49	1.14	1.94	0.004	1.24	0.84	1.83	0.29	1.67	1.16	2.41	0.006
Positive on the distal margin	1.54	1.19	1.99	0.001	1.53	1.00	2.34	0.051	1.57	1.13	2.17	0.007
Positive on the proximal and distal margin	0.95	0.52	1.72	0.85	0.70	0.17	2.82	0.62	1.10	0.57	2.14	0.77
*HER2 score*												
0 (-)	1.00				1.00				1.00			
1 (+)	0.97	0.85	1.10	0.62	0.82	0.67	1.01	0.06	1.08	0.91	1.29	0.37
2 (++)	0.92	0.76	1.11	0.39	0.80	0.61	1.04	0.09	1.02	0.78	1.33	0.88
3 (+++)	1.16	0.93	1.45	0.19	1.13	0.84	1.52	0.43	1.15	0.82	1.61	0.42
Unknown	1.33	1.17	1.50	<0.001	1.19	0.98	1.45	0.07	1.44	1.23	1.70	<0.001
*Linitis plastica*												
No	1.00				1.00				1.00			
Yes	1.37	1.01	1.86	0.045	2.13	1.25	3.65	0.006	1.18	0.81	1.73	0.38
*Borrmann classification*												
Borrmann I	1.00				1.00				1.00			
Borrmann II	0.92	0.80	1.07	0.30	0.87	0.72	1.06	0.17	0.99	0.78	1.25	0.93
Borrmann III	0.98	0.84	1.14	0.75	0.90	0.74	1.10	0.29	1.06	0.83	1.35	0.62
Borrmann IV	1.44	1.21	1.71	<0.001	1.44	1.12	1.85	0.005	1.48	1.14	1.93	0.003
Unknown	1.12	0.95	1.32	0.17	1.05	0.84	1.32	0.68	1.19	0.93	1.52	0.17
*Site*												
PGC	1.00											
DGC	0.94	0.88	1.00	0.058								

## Data Availability

The data used to support the findings of this study are included within the article in Tables 1, 2, and 3.
